# C‐type lectin domain family 5, member A (CLEC5A, MDL‐1) promotes brain glioblastoma tumorigenesis by regulating PI3K/Akt signalling

**DOI:** 10.1111/cpr.12584

**Published:** 2019-03-04

**Authors:** Hong‐Wei Fan, Qi Ni, Ya‐Ni Fan, Zhi‐Xiang Ma, Ying‐Bin Li

**Affiliations:** ^1^ Department of Clinical Pharmacology Lab Nanjing First Hospital, Nanjing Medical University Nanjing Jiangsu China; ^2^ Department of Neurosurgery the Second Affiliated Hospital of Nanjing Medical University Nanjing Jiangsu China

**Keywords:** brain glioblastoma, CLEC5A, PI3K/Akt pathway, tumorigenesis

## Abstract

**Objectives:**

Glioblastoma is the most common malignant glioma of all brain tumours. It is difficult to treat because of its poor response to chemotherapy and radiotherapy and high recurrence rate after treatment. The aetiology of glioblastoma is a result of disorders of multiple factors. Depending on cell signal transduction, these glioblastoma‐associated factors lead to cell proliferation, differentiation and apoptosis. Therefore, investigation of the potential factors which involved in the development of glioblastoma could provide a new target for the treatment of glioblastoma.

**Materials and methods:**

We analysed the transcript expression of CLEC5A in glioblastoma by accessing The Cancer Genome Atlas (TCGA). qRT‐PCR was performed to detect the RNA expression of genes in cells and tissues, and Western blot was used to measure the protein levels (Cyclin D1, Bcl‐2, BAX, PCNA, MMP2, MMP9, Akt and Akt phosphorylation) in tissues and cells. Cell proliferation, migration, invasion, cycle and apoptosis were measured by CCK‐8, transwell and flow cytometry assays, respectively. Ki67 level and lung metastasis were determined by immunochemistry and H&E staining.

**Results:**

In this study, we found that CLEC5A was highly upregulated in glioblastoma compared to normal brain tissues, which had an opposite relation with the overall patient survival. Downregulation of CLEC5A could inhibit cell proliferation, migration and invasion via promoting apoptosis and G1 arrest. In contrast, overexpression of CLEC5A stimulated cell proliferation, migration and invasion. In addition, we found that CLEC5A level was positively correlated with Akt phosphorylation level. Akt inhibitor or agonist could reverse the modulation effects of CLEC5A in glioblastoma. Moreover, In vivo results suggested that inhibition of CLEC5A significantly reduced tumour size, weight, cell proliferation ability and lung metastasis via inhibition of phosphorylation Akt.

**Conclusion:**

Both in vitro and in vivo evidences supported that CLEC5A was involved in glioblastoma pathogenesis via regulation of PI3K/Akt pathway. Thus, CLEC5A might serve as a potential therapeutic target in the treatment of glioblastoma in the future.

## 1 INTRODUCTION

Glioblastoma, WHO grade IV, is the most common malignant glioma of all the brain tumours and difficult to treat because of their poor response to chemotherapy and radiotherapy and with high recurrence rate after surgery.^1^, ^2^, ^3^ The average survival time of patients with glioblastoma is 9‐12 months, and the five‐year survival rate is less than 5% in adult.^4^, ^5^, ^6^ In addition, tumour cells infiltrate adjacent brain tissue, which often leads to failure of conventional treatment. Moreover, many chemotherapeutic drugs have been found to have serious side effects, which also affect the response of tumour treatment. About 30% of patients give up chemotherapy because of the serious side effects of treatment. Currently, effective treatment options are still lacking. Thus, investigation of effective therapeutic targets that involved the pathogenesis and progression of glioblastoma could further deepen the overall understanding of glioblastoma and would be expected to improve the clinical prognosis of patients with glioblastoma.

Proliferation and metastasis have always been the focus in the research of tumour pathogenesis.^7^ Like other malignant tumours, glioblastoma also exhibits abnormal cell proliferation and metastasis, which were caused by abnormal expression of a variety of genes.^8^, ^9^, ^10^, ^11^ C‐type lectin domain family 5 member A (CLEC5A), also known as C‐type lectin superfamily member 5 (CLECSF5) and myeloid DAP12‐associating lectin 1 (MDL1), is a C‐type lectin encoded by CLEC5A gene. This gene encodes a member of C‐type lectin/C‐type lectin‐like domain (CTL/CTLD) superfamily. Members of this family share a common protein fold pattern and have diverse functions, such as cell adhesion, cell‐cell signalling, glycoprotein turnover, and roles in inflammation and immune response. CLEC5A appears to be a member of a significant myeloid lineage activating pathway.^12^ However, the role of CLEC5A in glioblastoma remains unclear.

Phosphatidylinositol‐3‐kinase (PI3K)/Akt signalling pathway is widely involved in the regulation of cell proliferation, survival and differentiation, and other physiological and pathological activities.^13^, ^14^, ^15^ The mutation of upstream inhibitory factor PTEN, amplification of PI3K and over‐activation of Akt could lead to the disorder of PI3K/Akt signalling pathway. The occurrence of glioblastoma is frequently associated with activation of PI3K/Akt pathway.^16^ Increased level of phosphorylated Akt has been reported to associate with poor prognosis for glioblastoma patients.^17^, ^18^ EGFR amplification or overexpression, which leads to activation of PI3K/Akt signalling pathway, occurs in 40%‐50% of glioblastoma.^19^ Activating mutations in PIK3CA and PIK3R1, coding for subunits of PI3K, have been identified in approximate 10% of glioblastoma.^20^ Other stimulators of Akt activity, such as PDK1 and mTOR, were also upregulated in glioblastoma. Inhibition of PI3K/Akt pathway has been proposed to be a potential therapeutic strategy to treat glioblastoma.

The purpose of this study is to explore the regulation of the role of CLEC5A on the proliferation, apoptosis and metastasis of glioblastoma, and to further explore the related signalling pathway associated with CLEC5A in the pathogenesis of glioblastoma. This study could provide a theoretical basis for the prevention and treatment of glioblastoma.

## 2 MATERIALS AND METHODS

### 2.1 Cell lines, reagents and antibodies

Cell lines U87, A172, SHG‐44 and U251 were purchased from ATCC (Rockville, MD, USA). Akt inhibitor and activator were as following: AZD5363 (Selleckchem, Munich, Germany), IGF‐1 (R&D Systems, Minneapolis, MN, USA). Antibodies used in the present study were as following: CLEC5A (Abcam, Cambridge, MA, USA), Cyclin D1 (Abcam), PCNA (Abcam), MMP2 (CST, Danvers, MA, USA), MMP9 (CST), Bcl‐2 (CST), Bax (CST), phosphor‐Akt (CST), Akt (CST), GAPDH (Abcam) and β‐actin (Abcam).

### 2.2 Clinical samples

Thirty pairs of GBM tissues and adjacent normal tissues were obtained from patients with GBM in the Nanjing First Hospital from 5 January 2014 to 9 August 2017. These samples were collected in the process of surgical therapy. Written informed consent was obtained in all cases. The value of the adjacent normal tissue (first patient) was set as 1, and the relative expression of other adjacent tissues and GBM tissues was normalized to this tissue. All experimental protocols were approved by the Nanjing First Hospital Ethics Committee.

### 2.3 CLEC5A knockdown and overexpression

A lentiviral vector pLKO.1‐puro was modified to knockdown CLEC5A. Three oligonucleotides were designed to target the CLEC5A (shRNA1: GTTGTTGGAATGACCTTATT; shRNA2: CTGTGTTCAATGGCAATGTTA; shRNA3: CATTGGCCTAACAAAGACATT). Lentiviral plasmid was co‐transfected with packaging plasmids into 293T cells. Viral‐containing media were collected, filtered and concentrated. Knockdown efficiency was validated by Western blot and real‐time PCR. ShRNAs with highest efficiency were chosen for the function studies. To overexpress CLEC5A, plasmid vector pLenti 6.0 expressing the full length of CLEC5A was constructed (Genepharma, Shanghai, China). An empty plasmid vector was used as control. CLEC5A overexpression lentivirus was obtained by co‐transfecting plasmid into the 293T cells with the packaging plasmid. And then, the A172 and U87 cells were infected with lenti‐CLEC5A and control lentivirus. After 48 hours infection, cells were collected for infection efficiency measurement.

### 2.4 Western blotting

The tumour tissues or cells were lysed with RIPA buffer [50 mmol/L Tris (pH7.4), 150 mmol/L NaCl, 1% Triton X‐100, 1% sodium deoxy‐cholate, 0.1% SDS, 10 mmol/L NaF, 5 mmol/L EDTA, 1 mmol/L Na3VO4] containing protease inhibitors cocktail (Dalian Meilun Biotech Co., Ltd, Dalian, China). The supernatant was used for the following analysis. Protein concentration was determined by using BCA kit (Shanghai Yuan‐mu Biotech Co., Ltd, Shanghai, China). Equal amounts of total protein were loaded onto a 10% SDS‐PAGE gel and then transferred onto polyvinylidene fluoride membranes (PVDF, Dalian Meilun Biotech Co., Ltd, Liaoning, China). Subsequently, the PVDF membrane was blocked on 5% skim milk in TBST at room temperature for 1 hour and the membranes were incubated overnight at 4°C with primary antibodies. Then, the membranes were incubated with the appropriate HPR‐conjugated secondary antibody for 2 hours at room temperature. Next, the PVDF membranes were incubated with ECL reagent (Santa Cruz Biotechnology) in order to develop the blots.

### 2.5 RNA extraction and analysis

The total RNA was extracted by using TRIzol reagent (Invitrogen, Carlsbad, CA, USA). RNA samples were reverse transcribed using the TaqMan Reverse Transcription Kit (Invitrogen, Carlsbad, CA, USA). Real‐time PCR was performed as follows: firstly, 94°C for 4 minutes; secondly, 94°C for 30 seconds and 55°C for 30 seconds; and finally, 72°C for 45 seconds. Totally, there were 35 thermal cycles by using SYBR premix Ex Taq II kit (TaKaRa, Dalian, China) on an ABI 7900HT instrument (ABI, NY, USA). The PCR primer sequences: CLEC5A: Forward: 5’‐GTTTCACCACCACCAGGAGC‐3’; Reverse:5’‐GGCATTCTTCTCACAGATCC‐3’, GAPDH: Forward:5’‐GTTTCCTCGTCCCGTAG‐3’ Reverse:5’‐ATCTCCACTTTGCCACT‐3’. 2‐^ΔΔCT^ method was used to calculate the relative gene expression.

### 2.6 Microarray datasets

A total of 2 published microarray datasets containing survival information of glioblastoma patients were downloaded from the Array Express database (http://www.ebi.ac.uk/arrayexpress). GSE13041 and TCGA‐GBM were downloaded from The Cancer Genome Atlas (TCGA) (http://www.cancergenome.nih.gov). For survival analysis and cox regression, the level of each sample less than the median was regarded as CLEC5A‐low, and greater or equal to the median was defined as CLEC5A‐high. PFS and OS of each subtype were evaluated using TCGA‐GBM database. To evaluate the correlation between CLEC5A and cancer‐related pathways, gene‐set enrichment analysis (GSEA) was performed using GSE13041 dataset. Briefly, datasets and phenotype label files were created and loaded into GSEA software (v2.0.13). The number of permutations was set to 1000. A ranked‐list metric was generated by calculating the signal‐to‐noise ratio, which is based on the difference of means scaled according to the standard deviation (SD).

### 2.7 Cell Proliferation Assay

Cell proliferation assay was carried out based on a colorimetric assay using cell counting kit 8 (CCK8, Dojindo, Kumamoto, Japan) as described by the manufacturers. In brief, 100 μL cell suspension incubated for 0, 24, 48 and 72 hours was mixed with CCK‐8 solution and incubated for 1 hour and absorbance at OD450 was measured.

### 2.8 Apoptosis analysis

Cells (U251 and U87) cultured for 48 hours were washed twice with PBS and centrifuged at 1000 *g* for 5 minutes. The residue was resuspended with binding buffer (100 μL), and cells were stained with Annexin V (4 μL)/propidium iodide (PI, 3 μL) for 15 minutes in the dark at room temperature. After the incubation, 200 μL binding buffer was added and measured using FCM flow cytometry (BD, Bioscience, San Jose, CA, USA).

### 2.9 Cell cycle analysis

Cells that cultured to 75%‐80% confluence were washed with ice‐cold PBS, trypsinized and collected. The cells were then fixed in pre‐chilled 70% ethanol. After that, the cells were washed with PBS and stained in the dark with 4, 6‐diamidino‐2‐phenylindole (DAPI) (Thermo Fisher Scientific, Waltham, MA, USA) for 30 minutes at room temperature. The percentages of cells at different phases of the cell cycle were determined using CyFlow space flow cytometry from three independent experiments.

### 2.10 In vitro migration and invasion assay

In vitro cell migration assays were performed as described previously using transwell chambers (8 μmol/L pore size; Thermo Fisher Scientific). After 24 hours of serum starvation, cells were trypsinized and resuspended in serum‐free medium. Then, 2 × 10^5 ^cells were added to the upper chamber while complete medium was added to the bottom wells. Twenty‐four hrs later, cells that had migrated were fixed with 5% glutaraldehyde solution and stained with trypan blue to determine migrated cells. Images of 6 fields were captured from each membrane and the mean of 3 independent wells was used. As for cell invasion assay, the transwell membranes were pre‐coated with Matrigel (BD Biosciences). The experimental procedure of cell invasion assay was similar to that of the cell migration assay.

### 2.11 In vivo efficacy study

Eight male nude mice (Balb/c) aged 6‐8 weeks, weighing 18‐20 g, were purchased from Shanghai SLAC Laboratory Animal Co. Ltd., China, and housed under specific pathogen‐free (SPF) conditions (25°C‐27°C, 45%‐50% humidity, 12 hours/12 hours light/dark) at the centre of Nanjing Medical University Experimental Animals. The animals were randomly grouped into two groups. 200 μL U251 cells infected with shCLEC5A or its control virus at a density of 2.5 × 10^7^/mL were injected into the right abdominal flank. The mice were observed for tumour formation on a weekly basis. The tumour size was measured using calliper on a weekly basis for 5 weeks. Tumour volume was calculated using the formula: tumour volume = 0.5 × long diameter × short diameter^2^. All animal experiments were performed based on the guidelines approved by the Laboratory Animal Care and Use Committee of Nanjing Medical University.

### 2.12 In vivo tumour metastasis

Mice used in tumour metastasis assay were housed under same conditions as in tumour formation assay. A total of 5 × 10^6^ U251 with shCLEC5A or U87 cells CLEC5A overexpression were administrated in mice via tail vein injection. Eight weeks later, mice were sacrificed and lung tissues were collected. Lung metastasis was detected using H&E staining.^17^ Images of 6 fields were captured from each sample to eliminate the bias. The mice health conditions were observed weekly.

### 2.13 Data analysis

All statistical analysis was performed using the SAS statistical software, version 9.2 (SAS Institution Inc, Cary, NC), unless otherwise noted. Student's *t* test and one‐way ANOVA were used for comparing difference between two groups or multiple groups, respectively, and Pearson chi‐square test was used for categorical data analysis. Kaplan‐Meier survival analysis was used to plot the proportion of the population that were alive (Overall survival) by the length of follow‐up, in months. Hazard ratios (HR) with 95% confidence intervals (CI) were calculated using Cox proportional hazards regression analysis to examine the association of *CLEC5A* expression levels with patient survival. Two‐sided *P*‐value < 0.05 was considered statistically significant.

## 3 RESULTS

### 3.1 Differential expression of CLEC5A in brain glioblastoma

**Figure 1:**
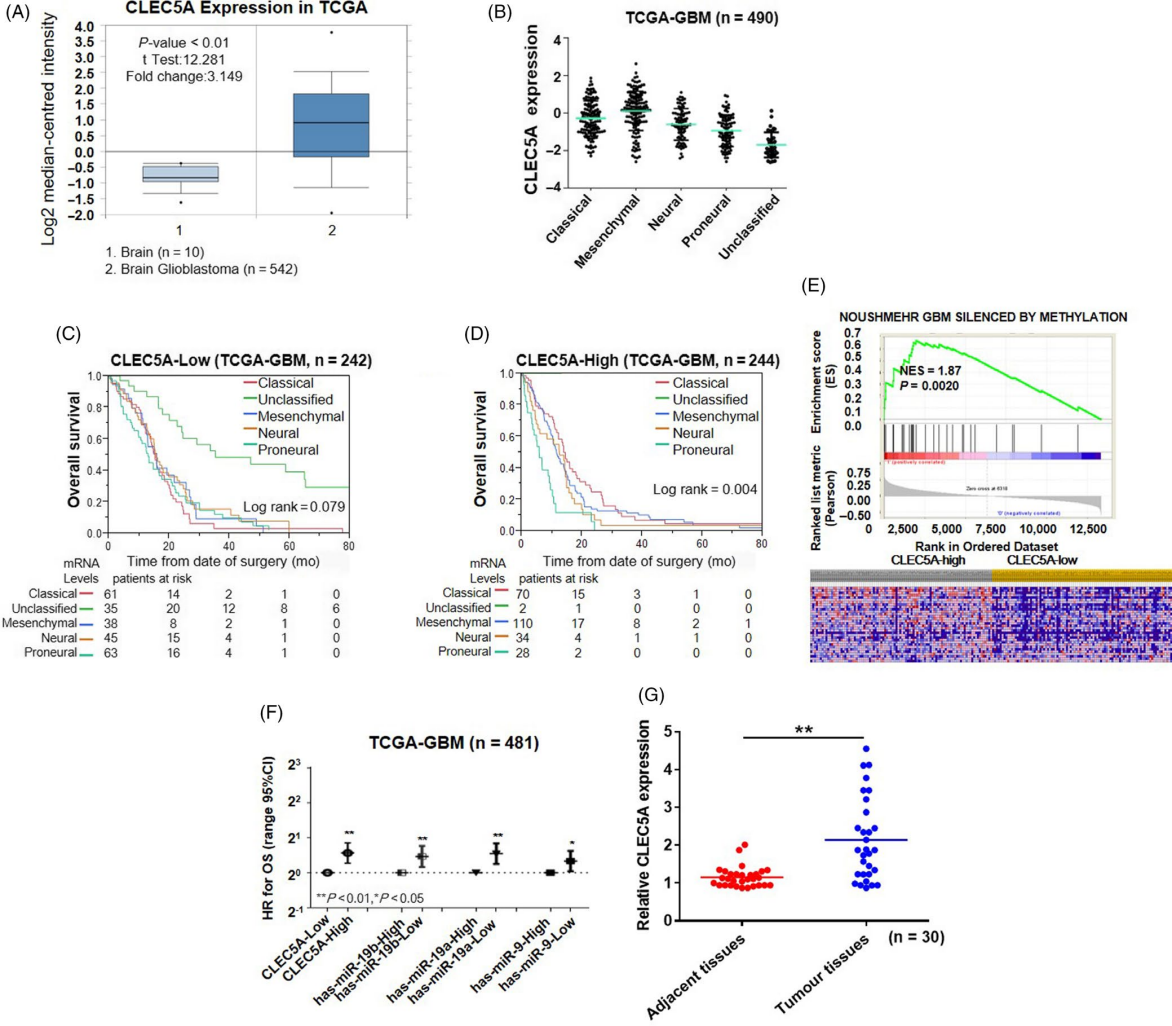
*CLEC5A* mRNA expression pattern in brain glioblastoma from public database. A, TCGA (n = 552) analysis of CLEC5A mRNA expression in brain glioblastoma tissues from brain patients and brain glioblastoma, showing *P*‐values <10‐4. The median‐centred intensity log2 expression values of *CLEC5A *were −0.826 in brain (n = 10) and 0.916 in brain glioblastoma (n = 542), *P* value was 6.08E‐9, *t* test was 12.281, fold change was 3.149. B, Kaplan‐Meier analysis for subtype of brain glioblastoma in TCGA‐GBM (log‐rank *P* = 0.079) (OS) of CLEC5A‐Low (C) and Log‐rank *P* = 0.0042 (OS) of CLEC5A‐High. D, *P* value calculated using one‐way ANOVA, Cox regression analysis for subtype expression. E, Enriched gene signatures associated with prognosis in CLEC5A‐high and CLEC5A‐low brain glioblastomas. F, Comparison of prognostic performance of CLEC5A and has‐miR‐19b, has‐miR‐19a, has‐miR‐9 with age, MGMT status and G‐CIMP status in TCGA‐GBM microRNA dataset. G, Comparison of CLEC5A mRNA expression in glioblastoma tissues (n = 30) and adjacent normal tissues (n = 30) from patients in our institutes

To determine whether *CLEC5A *mRNA expression is altered or co‐expressed in brain glioblastoma tissues, we searched multiple databases to compare *CLEC5A* expression levels involving brain tumour tissues and controls. TCGA (n = 552) (Figure 1A) and Sun Brain (n = 104) (data not shown) were selected to analyse *CLEC5A* mRNA expression in brain glioblastoma tissues. Results showed that the expression of *CLEC5A* mRNA in glioblastoma tissues was 3.149‐fold higher than that in normal brain tissues. Following this, we further analysed the expression pattern in different subtypes of glioblastoma. It was indicated that *CLEC5A* had the highest expression level in mesenchymal glioblastoma and lowest in unclassified subtype (Figure 1B). In addition, to further interrogate the overall survival (OS) in different glioblastoma subtypes from TCGA‐database, Kaplan‐Meier analyses were performed in both CLEC5A‐low and CLEC5A‐high sub‐cohorts. We found that in CLEC5A‐low glioblastoma sub‐group, unclassified group had a significant favourable OS than the other four subtypes (*P* = 0.04, Figure 1C). However, no significant OS difference be observed in different subtype groups of CLEC5A‐high glioblastomas (*P* = 0.079, Figure 1D). Moreover, gene‐set enrichment analysis was also determined to characterize the gene signature correlated with CLEC5A expression level in GBM, indicating the global DNA methylation levels between CLEC5A‐low and CLEC5A‐high tissues significant were significantly different (Figure 1E). In addition to epigenetic regulation on DNA methylation, microRNA also participate a profound role to modulate gene expression by targeting mRNA; thus, we analysed the microRNA expression patterns in TCGA‐GBM and concluded that has‐miR‐19 and has‐miR‐9 may have correlations responsible to CLEC5A expression (Figure 1F). The data mining from GBM databases demonstrated that CLEC5A had potential correlations with glioblastoma tumorigenesis.

A total of 30 pairs of glioblastoma tissues and adjacent normal tissues were obtained from glioblastoma patients from our institute. Consistent with conclusions from public database, *CLEC5A* mRNA expression in glioblastoma tissues was about 2‐fold higher than that in adjacent normal tissues from patients (Figure 1G), which further supported our observation of differential expression of CLEC5A in brain glioblastoma.

### 3.2 CLEC5A knockdown or overexpression in four glioblastoma cell lines

**Figure 2:**
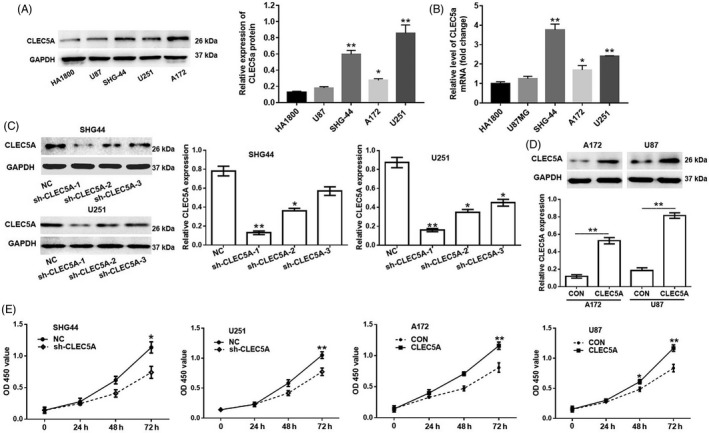
CLEC5A knockdown or overexpression in four glioblastoma cell lines. A, Relative CLEC5A protein level in one primary human foetal astrocyte cell line (HA1800) and four glioblastoma cell lines, GAPDH as loading control. B, Relative *CLEC5A* mRNA level in primary human foetal astrocyte cell line and glioblastoma cell lines, GAPDH as normalization. *P < 0.05, **P < 0.01 as compared to HA1800. C, Knocking down efficiency by lentivirus infection of three different shRNAs in SHG44 and U251. CLEC5A shRNA‐1 exhibits the best efficacy and was chosen in the following studies. *P < 0.05, **P < 0.01 as compared to HA1800. D, Overexpression of CLEC5A by lentivirus infection in A172 and U87. E, Cell proliferation assay using CCK‐8 kit in engineered cell lines with CLEC5A shRNA or CLEC5A overexpression virus. Values are expressed as means ±SD, *P < 0.05, **P < 0.01 as compared to the control

Based on the analysis from database indicating that CLEC5A had tightly correlation with glioblastoma, we next investigate the effect of CLEC5A on glioblastoma cell proliferation. One primary human foetal astrocyte cell line (HA1800) and four glioblastoma cell lines (SHG44, U251, U87 and A172) were selected. We found that SHG‐44 and U251 cells had higher expressed CLEC5A than U87 and A172, and HA1800 cells had the lowest CLEC5A protein level (Figure 2A). Consistently, quantitative real‐time PCR results also proved that SHG‐44 and U251 expressed much higher *CLEC5A *mRNA than U87 and A172, and HA1800 also had the lowest *CLEC5A *mRNA level (Figure 2B). According to the expression pattern in these cell lines, we modified CLEC5A expression level by knocking down with shRNA in U251 and SHG‐44 to inhibit CLEC5A with three different shRNAs (Figure 2C) while by over‐expressing CLEC5A using lentivirus in U87 and A172 (Figure 2D). The results of Western blot showed that shCLEC5A‐1 could inhibit endogenous CLEC5A protein level by more than 80% compared to scrambled shRNA both in U251 and SHG‐44. In contrast, the infected U87 and A172 with CLEC5A virus had increased CLEC5A protein level by 5‐ and 8‐fold, respectively. These results demonstrated that CLEC5A had a potential function in regulation glioblastoma cell proliferation. To further validate the effect of CLEC5A on glioblastoma cell proliferation, cell counting kit‐8 assay was conducted. The results indicated that inhibition of CLEC5A retarded cell growth, while upregulation of CLEC5A increased cell proliferation (Figure 2E). According to the efficacy of knockdown or overexpression, U251 and U87 cell lines were chosen for use in subsequent experiments.

### 3.3 CLEC5A regulated glioblastoma cell migration, invasion, apoptosis and cell cycle

**Figure 3:**
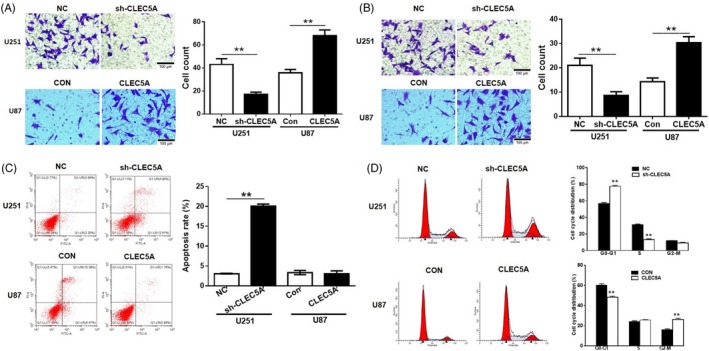
CLEC5A regulates cell proliferation in glioblastoma cells. A,B, Transwell assay for detection of migration and invasion in engineered cell lines with CLEC5A shRNA or overexpression. C, Apoptosis rates by flow cytometry assay in CLEC5A knocking down U251 and overexpressed U87, normalized to control cells. D, Cell cycle analysis using flow cytometry analysis in CLEC5A knocking down U251 and overexpressed U87 cells, normalized to control cells. Values are expressed as means ± SD, **P < 0.05 as compared to the control

Next, different assays were performed to investigate the functions of CLEC5A on glioblastoma cell migration, invasion, apoptosis and cell cycle. Cell proliferation assay CCK8 was also conducted. Consistently with previous transwell assay, inhibition of CLEC5A retarded cell growth while upregulation of CLEC5A increased cell proliferation (Figure 3A). Potential function of CLEC5A on modulation of cell proliferation led us to determine CLEC5A's role in cell apoptosis using flow cytometry method, and the FACS data showed that knocking down CLEC5A by shRNA could significantly increase cell apoptosis in U251 (Figure 3B). The outcomes of transwell assay reveal that knockdown of CLEC5A markedly decreased U251 cell migration and invasion, while overexpression of CLEC5A exerted opposite effect in U87 cells. In addition, the FACS data showed that knockdown of CLEC5A could significantly increase cell apoptosis in U251 (Figure 3C). However, overexpressed CLEC5A had mild amelioration of apoptosis in U87, which may due to the basal apoptosis level of control U87 is very low. Following these results, we next determined the cell cycle status in these cells, from which the results presented that overexpression of CLEC5A could promote U87 cells entering into S and M phase thus subsequently performing cell cycles for proliferation. On the contrary, inhibition of CLEC5A in U251 arrested more cells in G1 phase resulting in hurdle of cell cycles (Figure 3D). In summary, different approaches had revealed that CLEC5A regulated glioblastoma cell migration, invasion, apoptosis and cell cycle.

### 3.4 CLEC5A regulated glioblastoma tumorigenesis and metastasis via modulation various genes in vitro

There are various studies have been released in understanding pathways of glioblastoma migration and invasion. Among the complicated molecular mechanisms of brain tumour invasion, modification of receptor‐mediated adhesive properties of tumour cells, degradation and remodelling of extracellular matrix (ECM) have been proved to be one of the most important pathways.^21^ The matrix metalloproteinases are a family of zinc‐dependent endopeptidases which are involved in the degradation of extracellular matrix.^22^ MMP‐2 (gelatinase A) and MMP‐9 are type IV collagenase, which are especially important for the degradation of basal membrane.^23^ Proliferating nuclear antigen (PCNA) is a marker of cell proliferation and is expressed in the nuclei of cells during the DNA synthesis phase of the cell cycle.^24^

**Figure 4:**
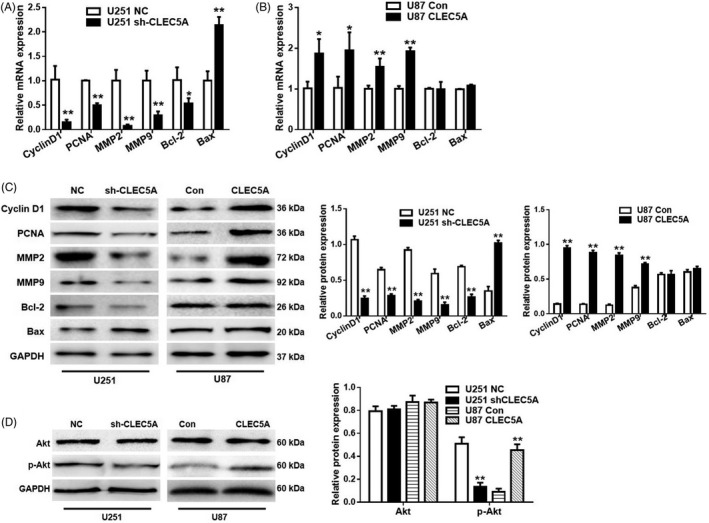
CLEC5A modulation on cell apoptosis genes expression and tumorigenesis. A, The mRNA levels of *Cyclin D1*, *PCNA*, *MMP2*, *MMP9*, *Bcl‐2* and *BAX* in U251 cells transfected with control or CLEC5A knocking down, normalized to GAPDH and control cells. B, The mRNA levels of *Cyclin D1*, *PCNA*, *MMP2*, *MMP9*, *Bcl‐2* and *BAX* in U87 cells transfected with control or CLEC5A overexpression virus, normalized to GAPDH. C, Protein level of Cyclin D1, PCNA, MMP2, MMP9, Bcl‐2 and BAX detected by Western blot in U251 cells with CLEC5A knockdown or U87 with CLEC5A overexpression, normalized to GAPDH. D, Total Akt and phosphorylated Akt protein level in U251 cells with CLEC5A knockdown or U87 with CLEC5A overexpression, normalized to GAPDH. Values are expressed as means ± SD, *P < 0.05, **P < 0.01 as compared to the control

According to the regulation function of CLEC5A in tumorigenesis, we examined the correlation of CLEC5A and extracellular matrix‐related genes *MMP‐2*, *MMP‐9* as well as apoptosis‐related genes *PCNA*, *Cyclin D1*, *BAX* and *Bcl‐2*. Quantitative real‐time PCR results showed that knockdown of CLEC5A could significantly inhibit *Cyclin D1*, *MMP‐2*, *MMP‐9*, *PCNA* and *Bcl‐2* mRNA levels in U251, while increased *Bax* gene expression (Figure 4A). On the other hand, overexpression of CLEC5A in U87 upregulated *Cyclin D1*, *MMP‐2*, *MMP‐9* and *PCNA* mRNA level, but had very limited effects on *Bax* and *Bcl‐2* gene (Figure 4B). Consistent results could be observed by Western blot, which further confirmed these proteins were regulated by CLEC5A in GBM (Figure 4C).

Another important pathway involved in tumorigenesis and cell apoptosis is PI3K/Akt pathway. Considering the correlation of Akt pathway and tumour cell proliferation, we consequently determined the modulation of CLEC5A on total Akt and phosphorylated Akt, which presented the endogenous activity of Akt pathway. It showed that CLEC5A level had a perfect positive correlation with AKT phosphorylation level but not total Akt (Figure 4D), in cohort with former ECM and apoptosis genes pattern. All above data proved that CLEC5A regulated genes expression involving ECM, apoptosis as well as PI3K/Akt pathways.

### 3.5 Akt inhibitor or agonist could reverse the modulation effects of CLEC5A in glioblastoma cells

According to the regulation of CLEC5A on Akt phosphorylation in glioblastoma cell lines, we further determined whether inhibition of Akt by Akt pathway inhibitors or activation of AKT by insulin signalling agonist could reverse the effects conducted by CLEC5A shRNA knockdown or overexpression. AZD5363, a novel pyrrolopyrimidine‐derived compound, inhibited all AKT isoforms and phosphorylation of AKT substrates in cells.^25^

**Figure 5:**
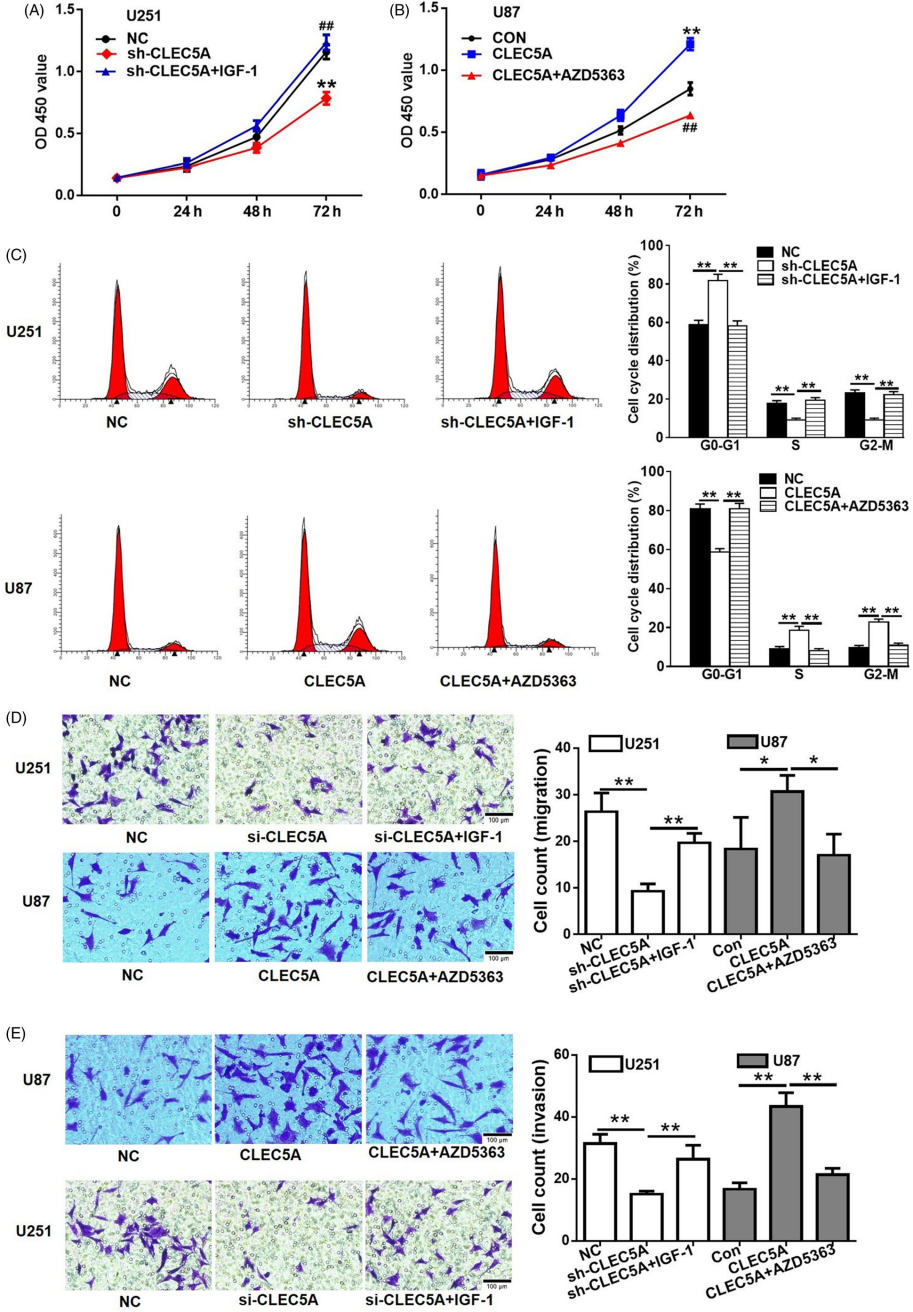
Akt agonist or inhibitor reversed the modulation effects of CLEC5A on glioblastoma cell lines, respectively. A, Anti‐proliferation effect of CLEC5A shRNA on U251 cell was reversed by Akt agonist IGF‐1. Cell growth was detected with CCK‐8. B, CLEC5A overexpression increased U87 cell proliferation, which was markedly attenuated by Akt inhibitor. C, Cell cycle modulation by Akt agonist in U251 cells with CLEC5A knockdown or inhibitor in U87 cells with CLEC5A overexpression, respectively. D, Cell migration modulation by Akt agonist in U251 cells with CLEC5A knockdown or inhibitor in U87 cells with CLEC5A overexpression, respectively. E, Cell invasion modulation by Akt agonist in U251 cells with CLEC5A knockdown or inhibitor in U87 cells with CLEC5A overexpression, respectively. Values are expressed as means ± SD, *P < 0.05, **P < 0.01

Our results revealed that activation of AKT pathway by IGF‐1 (10 nmol/L, 24 hours incubation), an insulin‐like growth factor, could revere anti‐proliferation effect of shCLEC5A on U251 cells (Figure 5A). In contrary, AZD5363 (2.5 µmol/L, 24 hours incubation) reduced the cell proliferation rate increased by overexpression of CLEC5A in U87 cells, which was even lower than control cells (Figure 5B). To explore the underlying mechanisms, we further examined the cell cycle status by FACS. The results showed that IGF‐1 attenuated the effect of shCLEC5A in U251 cells, while AZD5363 reversed the effects of CLEC5A overexpression by promoting cells entering into G0‐G1 phase in U87 cells (Figure 5C). These data strongly supported the hypothesis that CLEC5A functions via regulation of PI3K/Akt pathway. In addition, the data of transwell further indicating that IGF‐1 promoted metastasis rates in U251 with CLEC5A shRNA and AZD5363 weaken the metastasis ability induced by CLEC5A overexpression in U87 cells (Figure 5D, 5E). All of these results concluded that CLEC5A increased glioblastoma cell proliferation, which could be blocked by PI3K/Akt inhibitor.

### 3.6 Knockdown of CLEC5A significantly inhibited glioblastoma tumour growth in vivo

**Figure 6:**
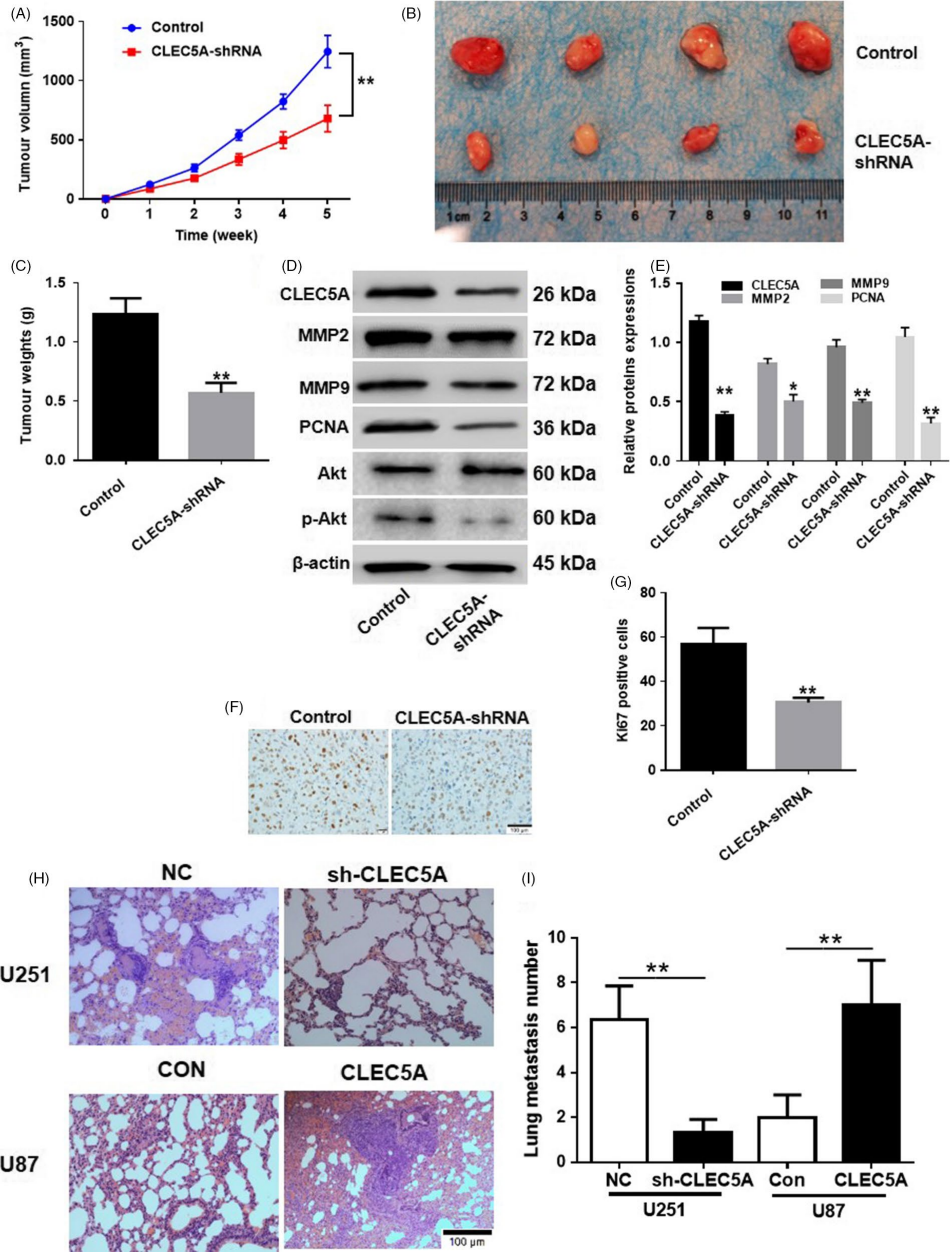
Knockdown of CLEC5A inhibited tumour growth in vivo. A, Time‐dependent tumour volume of cell transplantation in nude mice with shCLEC5A U251 normalized to control cells. B, After 35 d incubation of shCLEC5A transfected U251 cells in mice, the tumour was isolated and pictured. C, Quantification of tumour weight after 35 d of cell transplantation in nude mice with shCLEC5A U251 normalized to control cells. D, Protein levels of PCNA, MMP2, MMP9, Akt and Akt phosphorylation in tumour tissues were detected by Western blot. E, Quantification of Western blot of protein levels of PCNA, MMP2, MMP9, Akt and Akt phosphorylation. F and G, Immunochemistry staining and quantification of Ki67 in tumour sections. H and I, Lung metastasis staining and quantification on mice tail vein injection of CLEC5A knocking down U251 or overexpressed U87 cells, respectively. Values are expressed as means ± SD, *P < 0.05, **P < 0.01 as compared to the control

Based on the in vitro results, we further validated the role of CLEC5A in modulation of tumorigenesis in vivo by subcutaneous injection U251 cells containing shCLEC5A or scramble shRNA in nude mice and observed the tumour growth status (Figure 6A). After 35 days of injection, the tumours were assessed with volume (Figure 6B) and weight (Figure 6C). The in vivo results exhibited that inhibition of CLEC5A markedly inhibited tumour growth in mice, which was consistent with the in vitro studies. Immunochemistry staining of Ki‐67, a proliferation marker commonly used in pathological assessment, showed that tumour generated by shCLEC5A cells had lower level of Ki‐67 (Figure 6D,E), which further support the hypothesis that knockdown of CLEC5A inhibited tumour cell growth. In addition, we conducted tumour metastasis study by administration U251 cells containing shCLEC5A as well as U87 cells with lentivirus infected CLEC5A in cohort with control cells through tail vein injection and observed the lung metastasis level. The H&E staining of lung sections showed that mice injected with U251 (CLEC5A knockdown) had much lower metastasis level compared with control group (Figure 6F). In contrast, the mice injected with U87 (CLEC5A overexpression) exhibited more metastasis (Figure 6G), indicating the facilitating effect of CLEC5A on the metastasis of glioblastoma. Moreover, knockdown of CLEC5A downregulated protein level of MMP‐2, MMP‐9, PCNA and Akt phosphorylation in tumour tissues (Figure 6H,I). All in all, in vivo results were consistent with in vitro data and further confirmed the role of CLEC5A during glioblastoma tumorigenesis and metastasis.

## 4 DISCUSSION

Glioblastoma (GBM) is one of the most aggressive cancers with only less than 1‐year median survival in the general patient.^26^ Since the 1970 s, primary treatment has involved surgery followed by radiotherapy.^27^ Recently, targeted chemotherapy approaches using temozolomide have been used, although with modest effects on survival. Therefore, novel therapies are urgently needed. Insights into the genetic regulatory landscape of GB have been achieved through The Cancer Genome Atlas^28^ and identified abnormalities of several causal genes, including Idh1, Egfr.^29^ Furthermore, patterns of gene expression have been collated to identify molecular subgroups with putative prognostic or predictive significance.^30^ Here, we identified that CLEC5A is a critical protein linked with GBM pathology and clinical outcome.

CLEC5A, a type II transmembrane protein, was recently reported to have critical function in response to dengue virus,^31^ Japanese encephalitis virus^32^ and Listeria monocytogenes.^31^ Apart from these acute inflammatory diseases, CLEC5A was implicated in the progression of multiple chronic inflammatory diseases, such as autoimmune arthritis^33^ and chronic obstructive pulmonary disease (COPD).^31^ Several studies revealed that exaggerated cytokine released from CLEC5A‐expressing cells in respective pathologies. Here, we reported for the first time that CLEC5A was not only involved in the progression of glioblastoma (GBM) but also linked with clinical outcome. Using human malignant glioblastoma cell lines U251 and U87, which have differential expression of CLEC5A, our data demonstrated that CLEC5A expression was correlated with cell proliferation, apoptosis, migration and invasion. Moreover, in nude mice, CLEC5A knockdown significantly decreased tumour growth and metastasis. All these observations provided compelling evidences that CLEC5A was a critical player in tumorigenesis.

Previous reports suggested that CLEC5A was a critical pattern recognition receptor (PRR) which was activated by pathogen‐associated molecular patterns (PAMPs) expressed by microorganism. Moreover, activation of CLEC5A stimulated PI3K‐Akt pathway to enhance host immunity towards infection.^31^ Consistent with this, we observed that CLEC5A overexpression led to phosphorylation of Akt and promoted cell survival. Using AZD5363, a potent inhibitor of Akt with clinically proven efficacy in several tumours, we found both cell proliferation and metastasis were strongly inhibited. These findings identified that CLEC5A may function as a novel target for the therapeutic control of glioblastoma pathogenesis. However, the molecular mechanism is still need to be elucidated. Like most members of C‐type lectin, their exogenous and endogenous ligands have not been identified yet. One of research directions is to determine the ligands and binding partners of CLEC5A especially in the context of glioblastoma pathogenesis. In addition, an important hallmark of glioblastoma is intratumoral heterogeneity,^34^ suggesting multiple cell lineages coexist in tumour. Therefore, to dissect the functional role of CLEC5A on different cell types is of critical importance. For example, CLEC5A was reported to be expressed on microglia;^32^ more intriguingly, in the context of Japanese encephalitis virus infection, blockade of CLEC5A using peripheral administrated anti‐CLEC5A monoclonal antibody reduced the numbers of activated microglia and attenuate neuronal damage.

The major limitation of this study was the use of subcutaneous tumour model. Many studies have presented the limitations of these models, and the consensus among authors was that an orthotopic model is warranted. In this study, we have evaluated several orthotopic mouse glioma models, including transgenic mice (IDH1 R132H knocking), virus or chemical‐induced GBM model. However, the mutated genes are not specific to GBM but also contribute to other types of tumours, while the virus and chemical‐induced GBM models exhibit big individual variation. Thus, we chose the subcutaneous tumour model in our in vivo assay.

In conclusion, this study is of huge interest, because it suggested that CLEC5A mAb can cross blood‐brain barrier and might have clinical beneficial. Therefore, future studies will focus on the specific function of CLEC5A in glioblastoma pathogenesis in order to strength the hypothesis that CLEC5A serves as a therapeutic target for the treatment of glioblastoma.

## CONFLICTS OF INTEREST

The authors declare that they have no competing financial interests.
